# A survey about surgical hand antisepsis and implementation among surgeons in a newly-opened hospital in China

**DOI:** 10.1186/2047-2994-4-S1-P159

**Published:** 2015-06-16

**Authors:** S Xu

**Affiliations:** 1 West China Hospital Sichuan University, China

## Introduction

The study was performed in a newly opened hospital. It is expected to improve the quality of the hospital surgical hand antisepsis and to reduce the surgical site infection eventually.

## Objectives

To learn the surgeon’s knowledge level and implementation about surgical hand antisepsis, to provide guidance and decision-making basis in order to enhance the quality of surgical hand antisepsis further.

## Methods

The survey has been carried out in 11 surgical departments between September 17^th^ to 30^th^, 2014. The hospital infection control team (ICT) used a self-designed questionnaire to ask 100 surgeons to answer the questions. Contents of this survey included the general information about respondents, their knowledge level and implementation about surgical hand antisepsis, training attendance, whether further training required in the future. The survey’s quality control such as sample size, questionnaire modifications or adjustments, sample random selection has been done by ICT. The SPSS software10.0 has been used for statistics in this study.

## Results

1.96 copies of the survey questionnaire were returned. Male respondents accounted for 84.4%, less than 30 years old accounted for 59.4%, undergraduate surgeon accounted for 65.6%.

2. The average rate of the questions about surgical hand antisepsis answered correctly just was 68.6%, only 3.1% of respondents knew exactly the length of time when performing surgical hand antisepsis.

3. For the correct rate of the answer, the female surgeon answered correctly higher than male; the age <30 years group was higher than 30 to 39 age group and ≥50 age group; Postgraduate surgeon was higher than other categories.

4. From the departments, the general surgery, urology, neurosurgery and thoracic surgery, the correct rate of the answer were more than 70 percent.

5. The survey found that 80.2% of the total surveyed surgeon had accepted the training related to surgical hand antisepsis, 39.0 % attended the hospital-level’s training, 41.6% attended the department-level.

6. 83 % of respondents wanted to attend the further training about the knowledge related to surgical hand antisepsis and implementation.

## Conclusion

The surgeon’s knowledge level needs to be improved, it is necessary to carry out targeted standardized training regularly.

## Disclosure of interest

None declared.

